# Innovative Dementia Care Practices in Japan: Exploring Logical, Lateral, and Integrative Care Approaches

**DOI:** 10.1002/alz.086733

**Published:** 2025-01-09

**Authors:** Takahiro Tanaka, Kenichi Akune, Andrew Vernon

**Affiliations:** ^1^ Housenka Group / Lighthouse Health, Inc., New Rochelle, NY USA; ^2^ Housenka Group, Toyonaka‐shi, Osaka Japan; ^3^ Lighthouse Health, Inc., New Rochelle, NY USA

## Abstract

Japan’s aging demographic is witnessing a sharp rise in dementia cases, projected to reach 7.3 million by 2025 and 9.66 million by 2045. This increase not only foresees substantial economic strains, with social costs expected to hit 22.5 trillion yen by 2045 and 24.3 trillion yen by 2060, but also exerts pressure on the labor force as more individuals leave jobs for caregiving roles. Additionally, safety concerns are escalating with the rising number of people living with dementia reported missing. In response, innovative dementia care practices are essential.

The Housenka Group, based in Osaka, Japan, has pioneered three innovative approaches to dementia care: Logical Care (Fact Acceptance‐Based Aid), Lateral Care (Reality Affirmation‐Based Aid), and Integrative Care (Comprehensive Aid). Logical Care is centered around enhancing clarity and structure in communication to help people living with dementia understand and come to terms with their situation. A prime example of this method is seen when a daughter clearly explains to her mother the need to transition to a care facility. In contrast, Lateral Care is tailored for those people living with dementia who struggle to comprehend objective realities. It encourages caregivers to empathize with and validate the residents' personal perceptions of reality. A notable instance of this method in action involves caregivers addressing a resident using her former professional title, ‘President’, thereby significantly aiding her adjustment to a new environment.

Integrative Care blends elements of both Logical and Lateral Care, tailoring to individual requirements and behavioral responses. A significant implementation of this approach involved using pictograms to help a resident locate essential facilities, addressing issues like confusion and disorientation.

These approaches collectively aim to offer person‐centered, compassionate support to people living with dementia, valuing both their cognitive limitations and emotional well‐being. They provide a potential framework for effective dementia care applicable globally, especially in aging populations. The study highlights the critical need for flexible and understanding care practices in enhancing life quality for people living with dementia and their caregivers, marking a significant stride in dementia care innovation.

